# Newer antithrombotic drugs

**DOI:** 10.4103/0972-5229.76083

**Published:** 2010

**Authors:** Pranav Sikka, V. K. Bindra

**Affiliations:** **From:** Department of Pharmacology, ITS-CDSR, Muradnagar, Ghaziabad, Uttar Pradesh, India; 1 Department of Medicine, IDST, Meerut, Uttar Pradesh, India

**Keywords:** Antiplatelet drugs, antithrombotic drugs, thrombin inhibitors

## Abstract

Thromboembolic disorders are one of the disorders for which we are still on the look out for a safe and efficient drug. Despite the widespread use of antithrombotic drugs for the prevention and treatment of arterial and venous thrombosis, thromboembolic diseases continue to be a major cause of death and disability worldwide. This shows our inefficiency in searching efficacious and safe antithrombotic drugs. We have reached the basic mechanism of thrombus formation and by interrupting various steps of this mechanism, we can prevent as well as treat thromboembolic disorders. In continuation of Aspirin, now, we are using Clopidogrel, Ticlopidine and GpIIb/IIIa inhibitors (Abciximab, Tirofiban and Eptifibatide). Warfarin is an old antithrombotic drug which is still being used; but due to various side effects and drug interactions, we are bound to use newer drugs. Newer antiplatelet drugs include Prasugrel, Ticagrelor and Cangrelor, whereas newer thrombin inhibitors are Ximelgatran and Dabigatran. Apixaban is also a newer entry in this category as factor Xa inhibitor. Idrabiotaparinux is an indirect inhibitor of Xa as it accelerates the activity of antithrombin. Moreover, researches and trials for better and safe drugs are ongoing.

## Introduction

One of the major causes of morbidities and mortality worldwide is thrombolembolic disorder. Thrombosis is the process of formation of solid mass in circulation from constituents of flowing blood, and the mass itself is called as thrombus. Hemostatic plugs are the blood clots formed in healthy individuals at the site of bleeding, i.e., they are useful as they stop the escape of blood and plasma, whereas thrombi developing in the unruptured blood vessels may be harmful.

Virchow described three primary events which predispose to thrombus formation (Virchow’s Triad): alterations in or injury to the vascular endothelium, activation of the coagulation system, and/or reduction in flow.[1]

Thrombosis can be either arterial or venous. Both arterial and venous thrombi are composed of polymerized fibrin, platelets, trapped neutrophils and red blood cells. Arterial thrombi are commonly formed after endothelial injury due to rapidly flowing blood. Thus, platelets are abundant and fibrin is relatively sparse in arterial thrombus, whereas venous thrombus is formed due to blood stasis in veins, and thus, venous thrombus is mainly composed of fibrin and trapped RBC but less platelets. This fact is important to treat the patient effectively. Antithrombotic drugs are mainly of three types: (I) antiplatelet agents, (II) fibrinolytic drugs and (III) anticoagulants. By knowing the nature of thrombus, we can institute effective therapy, i.e., arterial thrombus should be treated by antiplatelet agents[2,3] and venous thrombosis should be treated by anticoagulants mainly[4,5] but they do not dissolve the clot that has already been formed. They prevent thrombus extension, recurrence and embolic complications. For the lysis of already formed thrombus, fibrinolytic drugs are used (Streptokinase, Urokinase, Alteplase, Reteplase, Tenectaplase, etc.)

Though antithrombotic drugs are widely used for the prevention and treatment of arterial and venous thrombosis, thromboembolic diseases are still the major cause of death and disability worldwide. Thus, it indicates that the available drugs are not very efficacious and sufficient to combat these disorders.[6] Here, we are going to review the older drugs in short, their shortcomings, i.e., unmet medical needs of currently available antithrombotic therapy and finally the newer drugs, newer targets, opportunities and challenges for them.

## The Unmet Medical Needs of Current Antithrombotic Therapy

Recurrent ischemic attacks are quite common even if a patient with previous history of stroke takes Aspirin/Clopidogrel/Ticlopidine or their combination, i.e., at present, the most widely prescribed antiplatelet agents are not efficient enough to prevent further attacks. This can be due to either resistance to these drugs or incomplete suppression of platelets by these drugs.

The only oral anticoagulant available in the market for more than half a century, i.e., Warfarin, also tells the same story with other limitations too. It has very slow onset of action for which it is combined with rapidly acting parenteral anticoagulants for the first few days. Due to some genetic polymorphism, dose adjustment is needed in almost every individual because of variable metabolism of Warfarin. There is one important problem of multiple drug interactions, and more than this, its narrow therapeutic index. Because of these inconveniences, patient compliance is poor on long-term basis.

Combination therapy (more than one antithrombotic agent at a time) has resulted in declining rate of recurrent ischemia but the incidence of hemorrhage has increased.[6]

## Natural Anticoagulant Mechanisms

In healthy vasculature, platelets are maintained in an inactive state by nitric oxide (NO) and prostacyclin (PGI _2_) released by endothelial cells of blood vessels. Endothelial cells also produce ADPase, which degrades ADP (adenosine diphosphate) from activated platelets, thereby preventing further platelet aggregation.[7] PGI_2_ opposes action of thromboxane A_2_(TXA_2_) and thus inhibits platelet aggregation and release. Antithrombin III (AT-III; a plasma protein) blocks the action of factors XII, XI, IX, X and II. Protein C (a plasma protein) inactivates factor V and VIII not blocked by AT-III, and it also enhances the action of tissue-plasminogen activator (t-PA). Also, heparan sulfate (a proteoglycan related to heparin), synthesized by endothelial cells, enhances the activity of AT-III.[8]

Both hemostasis and thrombosis depend on three general components – the vascular wall, platelets and the coagulation cascade.

Platelets contain two specific types of granules. Alpha granules express adhesion molecule P selectin on their membranes and contain factor V, von Willebrand factor (vWF), platelet derived growth factor (PDGF), fibrinogen and transforming growth factor (TGFβ), whereas δ (delta) granules contain ADP, calcium, serotonin and histamine.[9]

Vascular injury leads to transient vasoconstriction, and finally, platelets are exposed to extracellular matrix (ECM) via vWF and undergo three reactions: (I) adhesion and shape change; (II) secretion (release reaction) and (III) aggregation.[9]

Adhesion of platelets to ECM is facilitated by the interaction of vWF (released from endothelium) and glycoprotein Ib (Gp Ib) receptors (present on platelets). This adhesion leads to change in shape of platelets and there is release of granules containing ADP (from dense or delta granules). ADP is a potent mediator of platelet aggregation[9] and augments further ADP release. Now, TXA_2_ is synthesized and released which is also a mediator of platelet aggregation. On one side, vessel wall disruption augments platelet activation and aggregation, and at the same time, tissue factor initiates coagulation (extrinsic coagulation pathway). Phospholipid complexes on platelets activate intrinsic coagulation pathway. Platelet adhesion involves vWF, whereas aggregation involves GP IIb-IIIa receptors (present on platelets) via fibrinogen.[9] Common coagulation pathway finally produces thrombin which not only converts fibrinogen to fibrin but also activates platelets. These fibrin strands, along with platelets, now form platelet-fibrin mesh[6] leading to formation of hemostatic plug or thrombus.

**Figure 1:**
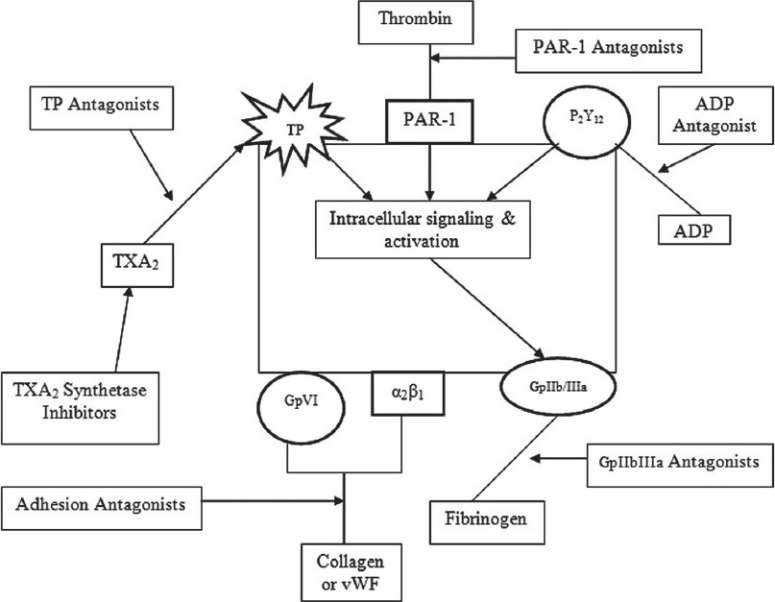
Site of action of various antiplatelet drugs. Platelet activation inhibitors include (i) TXA2 pathway inhibitors which block either synthesis or receptors of TXA2, (ii) ADP receptor (P2Y12) inhibitors and (iii) thrombin receptor (PAR-1) inhibitors. Adhesion antagonists block interaction of collagen and vWF with GpVI and α2β1 receptors on platelets. GpIIb/IIIa Inhibitors block binding of fibrinogen to their receptors on platelets. ADP, adenosine diphosphate; PAR-1, protease activated receptor 1; TP, thromboxane receptor; TXA2, thromboxane A2; vWF, von Willebrand factor

Antiplatelet drugs are classified on the basis of their site of action, i.e., drug that inhibit (i) platelets’ adhesion, (ii) platelets’ activation, (iii) platelets’ aggregation, and (iv) platelet mediated links with inflammation[6] [Figure 1].

## Drugs Inhibiting Platelet Adhesion

Platelet adhesion can be inhibited by interfering with the interaction between GP Ib (platelet receptor) and collagen or vWF. Various clinical trials are ongoing for the drugs acting through the above mechanism but none has reached phase III yet. Various strategies for inhibiting the ECM–platelet interaction include humanized monoclonal antibodies and aptamers against the receptors, small molecule peptide inhibitors and proteins derived from the medicinal leech.[6]

## Drugs Inhibiting Platelet Activation

Platelet activation can be inhibited by inhibiting TXA_2_ pathway, ADP pathway, thrombin and phosphodiesterase (PDE).

### 

#### Drugs inhibiting TXA2 pathway

Low dose (75–325 mg) Aspirin inhibits cycloxygenase-1 (COX-1) in such a way that only TXA_2_ production is inhibited and not of PGI_2_. Gastrointestinal tract (GIT) bleed, drug interactions and resistance are major drawbacks of Aspirin. To avoid these drug reactions, work is ongoing for new strategies such as inhibition of thromboxane synthetase enzyme and blockade of TXA_2_ receptors.[10] TXA_2_ synthetase is not much efficacious clinically because blockade of this enzyme results in accumulation of endoperoxide precursors which themselves are platelet agonists.[11]

#### Drugs inhibiting adenosine diphosphate pathway

Clopidogrel and Ticlopidine irreversibly inhibit ADP receptors on platelets. Actually, ADP activates ADP receptors (P_2_ Y_1_ and P_2_ Y_12_ receptors) to induce change in shape of platelets for their aggregation (P_2_ Y_12_ effect) and to increase their adhesiveness (P_2_ Y_12_effect). So, P_2_ Y_1_ and P_2_ Y_12_ inactivation will disfavor platelet aggregation.[12]

But (i) ceiling effect, (ii) resistance (genetic polymorphism), (iii) drug interactions (drugs metabolized by CYP3A4) and (iv) delayed onset of action are the major drawbacks of Clopidogrel. Ticlopidine is also not in use because of increased incidence of bleeding and serious neutropenia.

Newer drugs in this category are Prasugrel, Ticagrelor, Cangrelor and Elinogrel. Prasugrel produces its metabolite more efficiently, thereby leading to more rapid, consistent and potent inhibition of ADP-induced platelet aggregation than Clopidogrel does.[13] Drug interactions and resistance are not seen with Prasugrel. Onset of action is just 1–2 hours. But it has caused more bleeding[14] in some trials and should be cautiously given in patients with past history of stroke, age >75 years and weight <60 kg.

Ticagrelor, an orally active reversible P_2_ Y_12_ inhibitor, provides more rapid and complete antiplatelet action than Clopidogrel, whereas Cangrelor, also a reversible inhibitor of P_2_ Y_12,_ has rapid onset and offset of action but is administered intravenously (IV)_._ It is still in phase III of clinical trial. They all are being studied for prevention and treatment of ST elevation myocardial infarction (STEMI) and non ST elevation myocardial infarction (NSTEMI) following percutaneous intervention (PCI).

Elinogrel is a fast, potent, direct-acting (i.e., non-prodrug), selective, competitive, and reversible P_2_ Y_12_ inhibitor available in both intravenous and oral formulations.[15] In phase I clinical trial, Elinogrel demonstrated a rapid and potent inhibition of ADP-mediated platelet response, even in patients with coronary artery disease, who were deemed non-responsive to clopidogrel.[16] The ERASE MI trial, a pilot, phase IIA, randomized, double-blind, placebo-controlled, dose-escalation study, was designed to evaluate the safety and tolerability of escalating doses (10, 20, 40, and 60 mg) of Elinogrel as an adjunctive therapy for primary PCI for STEMI.[17] INNOVATE-PCI, a phase IIb clinical trial, was initiated in December 2008 to explore the compound’s clinical efficacy, biological activity, tolerability and safety.[15] In August 2010, its data were presented during the Hot Line II – coronary artery disease late-breaker session at the European Society of Cardiology congress in Stockholm and the findings establish the basis for clinical dose selection for a pivotal phase III program, which is anticipated to start in the first quarter of 2011.

#### Drugs inhibiting PAR-1

Protease activated receptors 1 and 2 (PAR-1 and 2) are receptors by which thrombin acts for platelet aggregation. PAR-1 antagonists such as SCH530348 and E5555 are under clinical evaluation.[18] SCH530348, an orally active PAR-1 inhibitor, is under phase III and E5555 is undergoing phase II evaluation.

#### Drugs inhibiting phosphodiesterase

Dipyridamole and Cilostazol are PDE inhibitors. PGI_2_acts through cAMP (as second messenger). Thus, more the cAMP, more will be the antiplatelet effect, but PDE degrades cAMP to form 5’AMP. So, PDE inhibitors tend to retain the amount of cAMP. They are being evaluated for their ability to prevent atherosclerotic diseases.

## Drugs Inhibiting Platelet Aggregation

Tirofiban, Eptifibatide and Abciximab are Gp IIb/IIIa inhibitors. Abciximab inhibits not only Gp IIb/IIIa but also α IIb/β_3_ receptors (for vWF) on platelets, thereby decreasing aggregation through fibrinogen.[19] It is given intravenously and has shown its efficacy in reducing ischemic events in the management of acute coronary syndrome (ACS) and as adjunctive therapy during PCI,[6] but trials with orally administered Gp IIb/IIIa inhibitors have failed to demonstrate any benefit. Moreover, pooled data have shown them to significantly increase mortality in ACS cases.[20] Reason behind this is unknown but may be related to partial agonistic activity and/or proinflammatory effects.[21] Therefore, development of this class of drugs has been halted following these disappointing results.[6]

## Drugs Inhibiting Platelet Dependent Inflammatory Pathways

Inflammation is an important determinant of progression of atherosclerosis and post-thrombotic syndrome which complicates deep vein thrombosis (DVT)[22] through CD40/CD40 ligand pathway and P selectin. Platelets also play an important role in inflammation.[23] CD40, released from activated platelets, acts as a cell attractant molecule and starts proinflammatory response. As it is highly expressed in atheromatous plaques also, inhibitors of CD40/CD40 ligand pathway can be novel molecules for delaying progression of atherosclerosis.[24]

P selectin molecule helps in the formation of platelet-leukocytes aggregates. Inhibition of P selectin will attenuate thrombus stimulus and formation. It has been proved in animal models of DVT[25] but is yet to be proved in humans.

## Present Anticoagulants and Their Future Prospects

Oral anticoagulants, i.e., Warfarin, is in use for more than half a century but too many adverse effects push for developments in this field. Drug interactions, very slow onset of action and variable response are major and problematic limitations of Warfarin.

Now, we are targeting toward inhibition of thrombin and other coagulation factors, especially Xa.

### 

#### Thrombin inhibitors

Thrombin is the most potent platelet agonist; it converts fibrinogen to fibrin and also amplifies its own production. Because of its multiple roles in coagulation, thrombin inhibitors not only block fibrin formation but also attenuate further thrombin formation and platelet activation.[26] The first direct thrombin inhibitor, Ximelgatran, has shown its efficacy in atrial fibrillation. The major drawback due to which it has been withdrawn is hepatotoxicity.[27] Latest addition in this series is Dabigatran etexilate. Being a prodrug, it is converted to Dabigatran by esterases after oral administration. Its half-life (*T*_1/2_) is 14–17 hours and is excreted out through kidneys; thus, once or twice daily administration is enough. It is undergoing phase III trial for prevention and treatment of venous thromboembolism (VTE) and for stroke prevention in atrial fibrillation. Other trials include comparison between Dabigatran and Warfarin (RE-LY) and two doses of Dabigatran with Warfarin (Re-COVER). Pooled data show no evidence of hepatotoxicity but Dabigatran level increases when used with P-glycoprotein inhibitors (Quinidine, Clarithromycin and Verapamil).[28,29]

#### Factor Xa inhibitors

It has been shown on animal models that upstream inhibition at the level of factor Xa causes less bleeding than downstream blockade of thrombin, but experiments in humans have not yet been performed.[30] High oral bioavailability, rapid onset of action, *T*_1/2_ of 7–15 hours and both renal and extrarenal excretion are few advantages. Rivaroxaban and Apixaban, oral factor Xa inhibitors, are undergoing phase III trial for VTE prophylaxis after elective hip and knee replacement. Rivaroxaban has been licensed in Europe and Canada for the above indications as studies have shown it to be more efficacious and with decreased risk of major bleeding.[31–34] Apixaban has been shown to be effective for thromboprophylaxis after orthopedic surgery.[35] Edoxaban is a new oral direct factor Xa inhibitor. Raskob *et al*. evaluated the efficacy and safety of different doses of Edoxaban and found it to be effective for preventing venous thromboembolism after total hip replacement.[36] Weitz *et al*. compared the safety of four fixed-dose regimens of Edoxaban with Warfarin in patients with non-valvular atrial fibrillation (AF) and found no significant differences in hepatic enzyme elevations or bilirubin values among the groups. The safety profiles of Edoxaban 30 and 60 mg qid in patients with AF were similar to that of Warfarin. In contrast, the Edoxaban bid regimens were associated with more bleeding than Warfarin.[37]

Idrabiotaparinux is a unique, long-acting pentasaccharide which acts by binding to antithrombin, thereby causing some conformational change that accelerates the rate at which antithrombin inhibits factor Xa. So, Idrabiotaparinux is an indirect inhibitor of factor Xa.[38] It is given once a week by subcutaneous route and is the only newer anticoagulant to have specific antidote.

## Monitoring of Antithrombotic Activity of Drugs

The prothrombin time (PT) is the laboratory test of choice for monitoring the anticoagulation status of patients treated with oral anticoagulants. It evaluates the extrinsic pathway factors but marked regional differences in sensitivity of thromboplastin used in the test lead us to use International Normalized Ratio (INR). The difference between the antithrombotic and anticoagulant effects of oral anticoagulants needs to be understood and applied in practice. The earliest changes in INR, showing anticoagulant activity, is typically noted 24–36 hours after drug administration (due to clearance of factor VII), but antithrombotic activity is not present until 5^th^ day of therapy which is due to clearance of factor II. This shows that loading dose of oral anticoagulants should be avoided because of increased risk of bleeding (severe reduction in factor VII) and risk of potentiation of hypercoagulable state (severe depletion of protein C). Most published studies indicate that an INR of at least 2.0 is required for effective anticoagulation. The risk of bleeding increases with increasing INR (>4.5–5.0). When oral anticoagulants are started for the first time, INR should be performed on a daily basis until it comes within therapeutic range for at least two consecutive days. Then, frequency can be decreased to one to two times a week for the next 2 weeks and then, once every 4–6 weeks if the patient is stable. Activated partial thromboplastin time (aPTT) test is done for monitoring the effect of parenteral anticoagulants, i.e., heparin (up to 1.5–2.5 times of control). The new agents do not affect the traditional clot-based PT/INR and aPTT tests, and monitoring and standardization require the development of new methods. In addition to clot-based assays, chromogenic assays, enzyme-linked immunosorbent assay (ELISA), high-performance liquid chromatography (HPLC), flow cytometry, and other techniques have been used to monitor these new drugs. On the other hand, some of the new antithrombotic drugs do affect the PT, aPTT, and activated clotting time (ACT); however, they behave differently from the Warfarin derivatives and heparin. Although the new antithrombotic drugs have been approved for clinical use, assay systems for monitoring most of them are still in development or have not been clinically validated.[39]

## Combination of Antithrombotic Drugs

The antithrombotic treatment of coronary artery disease is becoming increasingly complex. Anticoagulants and antiplatelet drugs are key therapeutic agents in the treatment of cardiovascular diseases. Given different mechanisms of action, combining these agents holds the potential for additive and perhaps even synergistic reductions in thromboembolic morbidity and mortality.[40] However, drug–drug interactions may lead to a greatly increased risk of gastrointestinal bleeding when these drugs are combined. Aspirin is often combined with more potent antiplatelet agents such as thienopyridines and glycoprotein IIb/IIIa inhibitors. With increasing severity of the coronary condition, the net benefit generally prevails even with an increasing number of antithrombotic drugs combined. However, as the patient slowly stabilizes after appropriate interventions, it is necessary to de-escalate the treatment in accordance with decreasing net benefit of prolonged combination therapy.[41] Jaoseph et al. conducted a population-based retrospective case control study from 2000 to 2005 and concluded that drug combination involving Aspirin with Clopidogrel or Warfarin was associated with a greater risk of gastrointestinal bleeding than that observed with each drug alone. Physicians should be aware of these risks to better assess their patients’ therapeutic risk–benefit profiles. Clopidogrel plus Aspirin combination had an efficacy nearly equivalent to Aspirin (CHARISMA study), Clopidogrel (MATCH study) and Warfarin (ACTICE-W study) when each was used alone in preventing cardiovascular events, but combination resulted in significantly increased risk of bleeding.[42] Leon et al. compared the efficacy and safety of three antithrombotic drug regimens – Aspirin alone, Aspirin and Warfarin, and Aspirin and Ticlopidine – after coronary stenting. As compared with Aspirin alone and a combination of Aspirin and Warfarin, treatment with Aspirin and Ticlopidine resulted in a lower rate of stent thrombosis.[43] Since patients at high risk for bleeding include elderly patients, patients with a history of hemorrhage or recent trauma and/or surgery, and patients with severe renal or hepatic dysfunction, treatment with triple therapy or dual therapy with anticoagulants plus antiplatelets should be prescribed only after thorough individual risk assessment and careful consideration of the risk–benefit ratio.

## Reversal Strategies for Antithrombotic Agents

Management of antithrombotic medication toxicity requires varying management strategies depending upon severity of bleeding. A careful risk–benefit analysis must be undertaken on a case-by-case basis.

### 

#### Vitamin K antagonists

Patients with serious or life-threatening bleeding need more aggressive reversal of their coagulopathy. The reversal of Warfarin coagulopathy involves three principal measures: withholding Warfarin therapy, giving intravenous vitamin K (up to 10 mg slow infusion), and supplementing these interventions with fresh frozen plasma (FFP), 5–15 mg/kg IV; packed cell concentrate (PCC), or recombinant factor VIIa (rFVIIa), 15–90 µg/kg IV, depending on the urgency of the situation. The use of PCC can be considered for the treatment of hemophilia in patients who have developed inhibitors to single-factor concentrates. When there is not enough time for the thawing of FFP to treat life-threatening hemorrhage and PCC is unavailable, the use of rFVIIa should be considered as its effects are immediate. As a general rule, rFVIIa should be reserved for use as a rescue agent in patients with serious or life-threatening bleeding complications of antithrombotics, for whom conventional therapies have failed or are unavailable. The treatment may need to be repeated in cases of persistent coagulopathy despite initial interventions aimed at reversal.[44–48]

#### Heparin

Reversing anticoagulant effects of heparins involves two principal strategies: allowing the anticoagulant effect to dissipate over time or protamine administration. Protamine should be administered cautiously, 1 mg protamine for every 100 U of heparin, with a maximum intravenous dose of 50 mg over 10 minutes. There is currently no reliable antidote available for low molecular weight heparin (LMWH). Fortunately, however, bleeding complications related to LMWH are infrequent. Low molecular weight derivatives of protamine sulfate are in development and there are preliminary reports that these agents may also be effective as antidotes to LMWH. Heparinase is also under investigation as an antidote to heparin-induced bleeding, although it is unclear whether this approach will be effective as an antidote for LMWH or other indirect thrombin inhibitors.[49]

#### Antiplatelet agents

Available options include discontinuing the antiplatelet medication, administering desmopressin [deamino-d-arginine vasopressin (dDAVP)], and transfusing platelets. In addition, there is a potential role for rFVIIa in this setting.

#### Fibrinolytic agents

In patients with massive bleeding, hemodynamic compromise, or intracranial hemorrhage following fibrinolytic administration, all anticoagulant, antiplatelet, and fibrinolytic medications should be immediately discontinued. Cryoprecipitate may be utilized to replenish fibrinogen stores (particularly when fibrinogen levels are less than 100 mg/dl), and FFP may be administered to replete all coagulation factors.

## Alternative Antithrombotic Agents

Currently, there are no specific antidotes for bleeding in patients receiving novel antithrombotic agents. There is some literature to support the use of rFVIIa in patients with complications while on these medications, but further investigation is needed. Administration of activated PCCs reduces bleeding in Dabigatran treated animals. Although normal volunteer and *ex vivo* data suggest that rFVIIa antagonizes the anticoagulant effect of a variety of agents, particularly Dabigatran, there are no data demonstrating utility in actively bleeding patients. Recently, an active-site-blocked recombinant FXa mutant lacking the membrane-binding domain was shown to reverse the anticoagulant effects of FXa inhibitors *in vitro* and in animals by competing with endogenous FXa for the inhibitor.[50]

## Conclusion

We are using various antithrombotic drugs for more than half a century but not a single one is sufficiently efficacious. If some are efficacious, they produce side effects or onset of action is delayed. Every time there is fear of hemorrhage by excessive dose, i.e., dose titration and difference in patient to patient response pose a great problem in practicing these drugs. Moreover, type of thrombus is also important to treat the patient (either venous or arterial) because drugs for different thrombi are different and there should be proper clinical judgment before giving drugs. So, still we are in search of newer and better antithrombotic drugs but there are miles to go.
